# Supplementary material for: Gromov–Wasserstein unsupervised alignment reveals structural correspondences between the color similarity structures of humans and large language models
Source: Sci Rep. 2024 Jul 10;14:15917. doi: 10.1038/s41598-024-65604-1 (PMC11237038; doi:10.1038/s41598-024-65604-1)
Supplement: Supplementary file 1 — Supplementary Information. [file 41598_2024_65604_MOESM1_ESM.pdf]

93-color similarity structure of large language models can be aligned with that of humans via unsupervised alignment

Genji Kawakita*[1], Ariel Zeleznikow-Johnston†[2,3], Naotsugu Tsuchiya‡[2,3,4,5], and Masafumi Oizumi§[6]

[1]Department of Bioengineering, Imperial College London, London, UK
[2]School of Psychological Sciences, Monash University, Melbourne, Australia
[3]Turner Institute for Brain and Mental Health, Monash University, Melbourne, Australia
[4]Center for Information and Neural Networks (CiNet), National Institute of Information and Communications Technology (NICT), Osaka, Japan
[5]Department of Qualia Structure, ATR Computational Neuroscience Laboratories, Kyoto, Japan
[6]Graduate School of Arts and Science, The University of Tokyo, Tokyo, Japan

The PDF file includes:

Supplementary Text 1: Human color similarity judgment experiments
Supplementary Text 2: Unsupervised alignment with GPT-4 Vision
Supplementary Text 3: Comparison of supervised and unsupervised methods
Figure S1: Scatter plots of similarity ratings
Figure S2: Optimal transportation plans
Figure S3: Results of the optimization of ϵ (ϵ v.s. GWD)
Figure S4: Unsupervised alignment between the color similarity structure of the human color-neurotypical participants and that of GPT-4 Vision
Figure S5: Unsupervised alignment between the color similarity structure of GPT-4 and that of GPT-4 Vision
Movie S1: 3D MDS Embeddings

*Email: g.kawakita22@imperial.ac.uk
†Email: ariel.zeleznikow-johnston@monash.edu
‡Email: naotsugu.tsuchiya@monash.edu
§Email: c-oizumi@g.ecc.u-tokyo.ac.jp

Supplementary Text 1: Human color similarity judgment experiments

The data is derived from the same experiment as reported in [1].

Ethics

Experimental procedures were approved by the Monash University Human Research Ethics Committee (Project ID: 17674). Participants were provided electronically with written consent forms prior to the commencement of the experiment and provided electronic consent to participate. Participants were compensated for their time at a rate of £5.27 for an experimental duration of approximately 40 minutes.

Design

Participants Participants were recruited remotely through Prolific, an online participant recruitment platform. Participants accessed the experiment and provided data using their own personal computers. Only English native speakers were recruited. We recruited 488 general-population (neurotypical) and 360 self-identified color-atypical (atypical) participants prior to data cleaning.

Exclusion - General Participants who failed to meet the inclusion criteria were excluded from the analysis. Firstly, we removed participants who failed to complete the experiment. Secondly, we excluded participants with a catch score (see below) of $< 77\%$. Catch trials were included to ensure participant attention and scattered randomly among the main trials. Lastly, the experiment was designed as a 'double-pass paradigm', meaning participants performed each sequence of main trials twice. Participants whose responses across the two passes were not strongly correlated < 0.5 were excluded, as low 'double-pass' correlation is indicative of inattentive or neglectful responding [2, 3]. 62 out of 488 neurotypical participants were excluded, leaving 426 (87%) for the main analysis.

Exclusion - Color Atypical We collected a cohort of 548 participants who self-identified as color blind. In addition to the general exclusion criteria, these participants were also screened using a modified online Ishihara test. Participants viewed a set of 28 Ishihara color plates and were asked to report the number they observed. 16 of the plates were standard and used as a positive control, with participants excluded if they correctly identified $> 80\%$ of the plates (i.e. made fewer than three errors) [4]. 12 plates consisted of standard Ishihara plates that were red-shifted or blue-shifted so that the number should be correctly identifiable by participants with red-green color deficiencies [5]). These plates were used to detect participants who falsely identified as red-green color blind, with participants excluded if they correctly identified $< 80\%$. After these additional exclusion criteria, 257 of 548 (47%) participants who self-identified as color blind were used for the main analysis.

Display apparatus Due to the nature of online experimentation, participants used their own computer screen to perform the experiment. The stimuli for the current study were based on the color swatches used by [6]. This 93-color set was selected from the Practical color Co-ordinate System (PCCS), a color system developed by the Japan Color Research Institute. The PCCS organizes colors into 14 distinct "tone" categories, each of which includes 24 different hues. In this system, the term "tone" refers to a combination of metric lightness and metric chroma, representing a group of hues that produce a similar color perception. Colors within the same PCCS tone are perceived to have consistent lightness and chroma levels, regardless of their specific hue. All stimuli were presented as solid colored circles 120 pixels in diameter on a grey (#7F7F7F) background.

In contrast, the 23 colors used in the previous study [7] were an extension of a dataset originally consisting of 14 colors, taken from Ekman (1954) and reproduced in Shepard (1980). The original dataset comprised direct similarity judgments across a set of 14 colors with wavelengths ranging from 434 to 674 nanometers, with uniform luminance and near maximal saturation. The previous study [7] extended this dataset to 23 color stimuli by interpolating between the colors in the original dataset to achieve a denser coverage, but they did not give details of the nature of this extension.

While we do not have access to the exact hex codes used in the previous study[7], we infer that the distribution of colors across categories significantly differs between their 23-color set and our 93-color set, especially in luminance and saturation (presumably, [7] did not extend the original color sets in these two dimensions). The PCCS-based color set used in our study is designed to provide a systematic and balanced representation of colors across various tones and hues, ensuring a comprehensive coverage of the color space.

Procedure After recruitment through Prolific, participants were directed to the experiment hosted on Pavlovia. The first page of the experiment was a consent form that they could electronically sign by pressing the spacebar. Participants were informed that the data collection process was anonymous and that they could quit the experiment at any time. Following consent, participants were provided written instructions on how to complete the experiment. This was followed by 9 practice trials, seven of which were color similarity judgments and the rest were catch trials.

Main trials for neurotypical participants proceeded as follows. First, a fixation cross was presented in the centre of the screen for 250 ms. Following this, the two stimuli were presented as solid-colored circles for 250 ms. Considering the centre of the screen as the midpoint, each stimulus was presented 180° apart and at a radius of 8% of the width of their screen. The stimuli were randomly assigned to a position within ±30° of horizontal meridian in order to prevent retinal adaptation between trials. Lastly, the participants were presented with a response screen and were directed to select a specified value from 0 (most similar) to 7 (most dissimilar). After responding, participants were asked to click on the centre of the screen to initiate the next trial.

Atypical participants were presented with a slightly updated version of the same task. Instead of stimuli being presented randomly within ±30° of horizontal meridian, they were presented randomly in two out of four possible locations equidistant from the centre of the screen and maximally spaced from each other. Additionally, participants reported using values from -4 to $+4$ (with zero excluded) instead of 0 to 7. All other parameters remained the same. (Later, we transformed these values to 0 to 7 with a uniform distance of 1).

Catch trials involved no presentation of colored stimuli patches. Instead, participants were shown a response screen where they were prompted to click a specific number. All other aspects of the response screen were the same.

During practice trials, participants were provided feedback on what selection they made, consisting of both the value they selected and the text 'Very Similar', 'Similar', 'Different' or 'Very Different' for selections of 0/1, 2/3, 4/5, 6/7 respectively for the neurotypical participants, or $-4/-3$, $-2/-1$, 1/2, 3/4 for the atypical participants. At the cessation of these practice trials they are asked to press the SPACE button to proceed to the main trial set.

Following the practice trials, participants completed the main task. As with the practice trials, catch trials were randomly inserted among the main trials. Each participant was randomly allocated a set of color pairs out of the total 4371 unique pairs of 93 colors (including pairs of the same color), which were presented in a random sequence. Color-neurotypical participants were allocated 162 color pairs. After providing a response for each color pair once, color-neurotypical participants performed a repeat of the first 162 trials, identical in stimuli and sequence (double-pass). In total, this comprised of 324 main trials and 20 randomly interspersed catch trials. Color-atypical participants were allocated 81

color pairs, which were also presented in a double pass manner for a total of 162 main trials and 10 catch trials.

Supplementary Text 2: Unsupervised alignment with GPT4-Vision

To further investigate the influence of input modality on LLM performance, we collected additional data using the GPT-4 Vision model (`gpt-4-vision-preview`), which allows for the input of color patches as images. We provided GPT-4 Vision with pairs of color patches and asked the model to rate the dissimilarity between the colors using the following prompt:

> Rate the dissimilarity of the pair of colors in the two images on a scale of 0-7, with 0-1 being Very Similar, 2-3 being Similar, 4-5 being Different, and 6-7 being Very Different. Your rating should be any real number between 0 and 7. Your answer should be only the rating in the form of a number. No explanation is needed.

The temperature parameter was set to 0.7, consistent with the settings used for GPT-3.5 and GPT-4 with text input. We collected a complete set of similarity judgment responses for all possible pairs of 93 colors. The dissimilarity matrix of 93 colors from GPT-4 Vision was obtained directly from a single trial.

The Spearman correlation between the color dissimilarity matrix of GPT-4 Vision and that of color-neurotypical participants (Supplementary Fig. S4(a)) was found to be $\rho = 0.56$. Although this correlation is lower than those obtained with text input (GPT-4: $\rho = 0.77$, GPT-3.5: $\rho = 0.62$), the unsupervised alignment using GWOT revealed a striking similarity between the color similarity structures of GPT-4 Vision and color-neurotypical participants.

The top-1 matching rate between GPT-4 Vision and color-neurotypical participants was 59.1%, significantly higher than the chance level of 1.08% (Supplementary Fig. S4(b)). The optimal transportation matrix clearly shows that similar colors are matched with each other, indicating that GPT-4 Vision captures the overall color similarity structure in a manner comparable to color-neurotypical participants, even though its performance in capturing subtle differences between similar colors is not as strong as GPT-4 with text input (Supplementary Fig. S4(c)(d).

Furthermore, we compared the color similarity structures of GPT-4 and GPT-4 Vision directly (Supplementary Fig. S5). The Spearman correlation between their color dissimilarity matrices was found to be 0.58 (Supplementary Fig. S5(a)), and the top-1 matching rate was 52.7%(Supplementary Fig. S5(b)). These results suggest that while GPT-4 and GPT-4 Vision are not identical in their color similarity judgments, they share substantial structural similarities (Supplementary Fig. S5(c)(d).

Supplementary Text 3: Comparison of unsupervised and supervised alignment methods

In this study, we do not claim that unsupervised alignment methods, such as Gromov-Wasserstein Optimal Transport (GWOT), are superior to supervised alignment methods like correlation-based Representational Similarity Analysis (RSA) in all situations. These methods serve different purposes and should be used appropriately depending on the research question and available data.

Unsupervised alignment methods, like GWOT, are particularly useful when comparing similarity structures without relying on pre-defined label correspondences. They can reveal detailed structural correspondences by considering all possible item mappings. In contrast, supervised alignment methods, such as RSA or Procrustes alignment, are suitable when comparing similarity structures using pre-defined label correspondences. They provide a measure of similarity that does not distinguish alignment at the categorical and item level.

Both approaches have their merits, and the choice between them depends on the specific research question and the availability of label information. Here, we summarize the pros and cons of each approach:

Unsupervised alignment (e.g., GWOT):

- Pros: Reveals detailed structural correspondence between similarity structures. Can find the best alignment between two structures by considering all possibilities of correspondences.
- Cons: Computationally expensive. Needs to solve non-convex optimization, whose solution depends on initialization and hyperparameters.

Supervised alignment (e.g., Procrustes alignment or correlation-based RSA): - Pros: Provides an overall measure of coarse similarity between structures. Easy to compute and does not rely on non-convex optimization. - Cons: Cannot distinguish between categorical-level and fine-item-level alignment. Alignment is found only based on assumed correspondences between items (more constrained).

In our previous study[8], we conducted simulations to demonstrate that GWOT can capture differences in similarity structures even when the correlation remains constant ($\rho = 0.90$). The simulations considered three scenarios:

1. One-to-one fine “correct” mapping: GWOT reveals a close match between structures at the individual item level, consistent with the high correlation.
2. Many-to-many coarse mapping: Despite a high correlation, GWOT shows that the match is only at the coarse-categorical level, not at the individual item level.
3. Mapping different from assumed correspondences: GWOT reveals that the mapping between structures differs from the assumed correspondences, even though the correlation remains high.

These simulations highlight the qualitative differences between unsupervised and supervised comparison methods. While correlation-based RSA gives the same evaluation in all three cases, GWOT can distinguish between the different scenarios and provide a more detailed assessment of the structural correspondence between similarity structures.

Our intention is not to replace supervised alignment methods with unsupervised ones. Rather, it is to demonstrate that unsupervised methods can complement supervised methods by revealing more nuanced structural differences. The choice between these approaches depends on the specific research question and the availability of label information.

Supplementary Figure S1

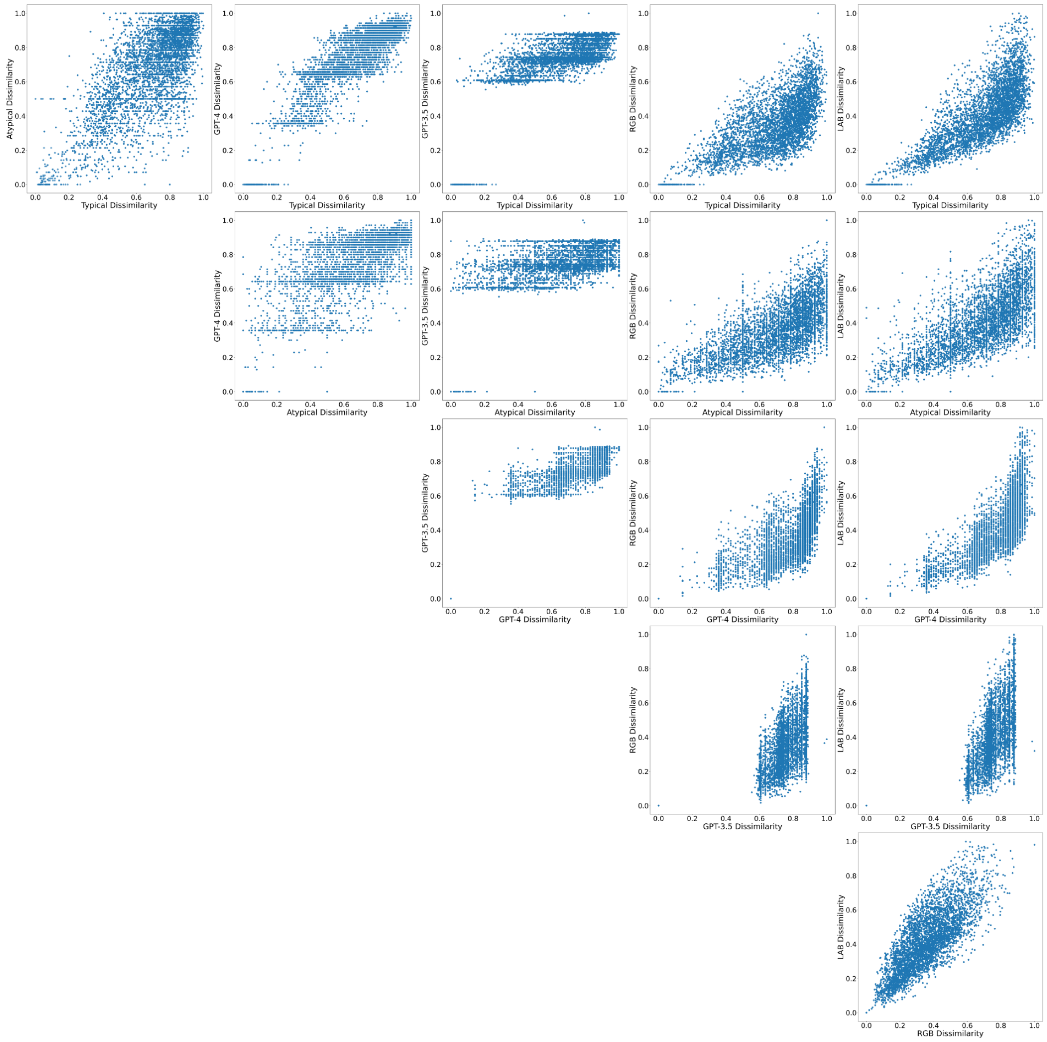

Figure S1: **Scatter plots of dissimilarities**. The scatter plots of dissimilarity of all pairs of the 93 colors. Each panel is the scatter plot between each participant group, GPT or models. The column and the row are arranged in the order of the color-neurotypical participants group, the color-atypical participants group, GPT-4, GPT-3.5, and the RGB and LAB color spaces.

Supplementary Figure S2

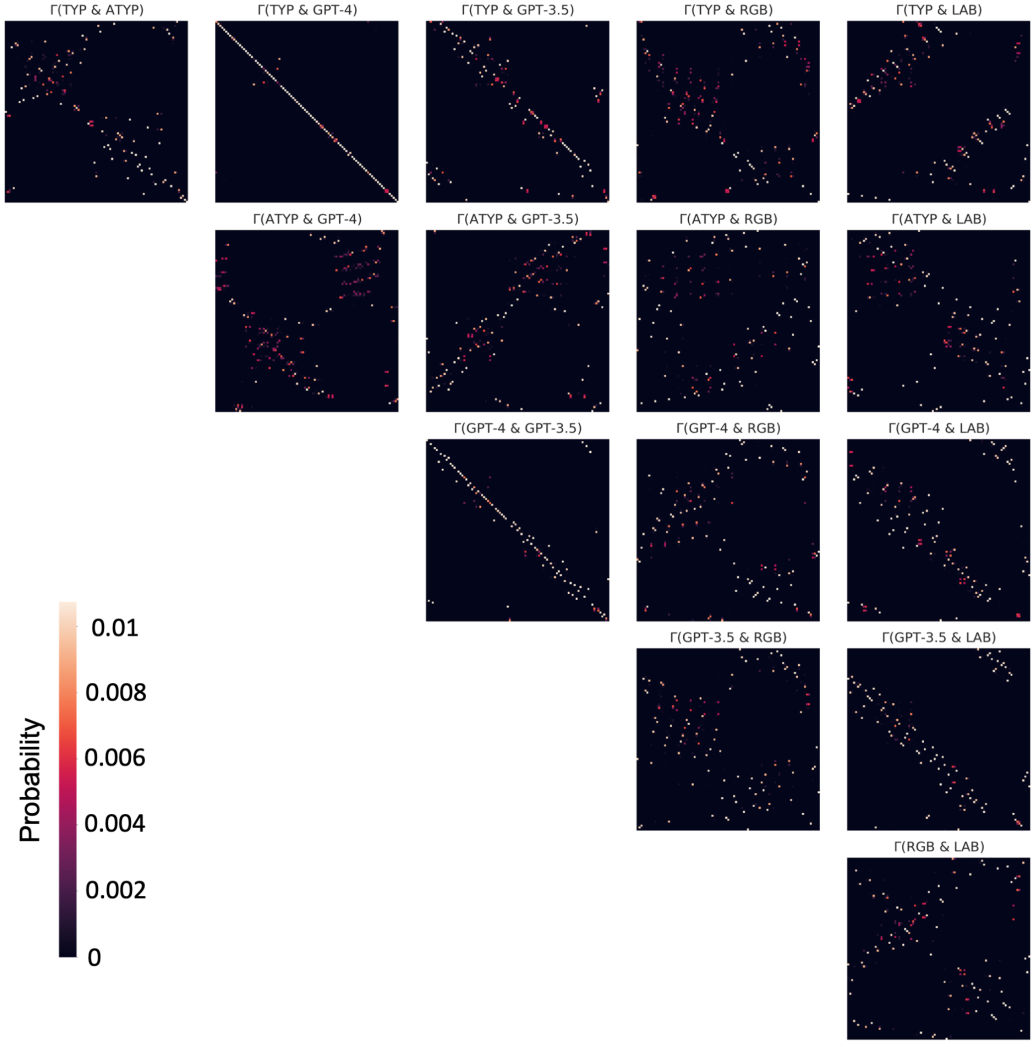

Figure S2: **Optimal transportation plans**. Optimal transportation plans Γ^* between the similarity matrices. Panels are arranged in the same way as Figure S1.

Supplementary Figure S3

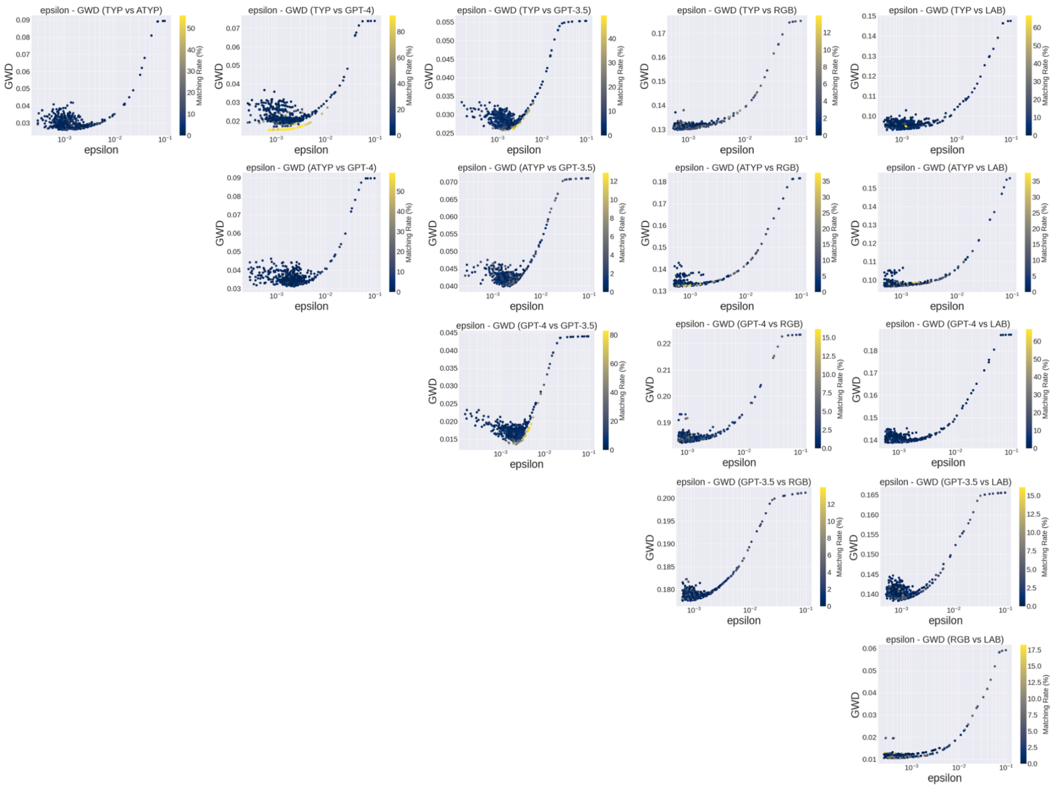

Figure S3: **Results of the optimization of ϵ.** The epsilon-GWD plots. Panels are arranged in the same way as Figure S1.

Supplementary Figure S4

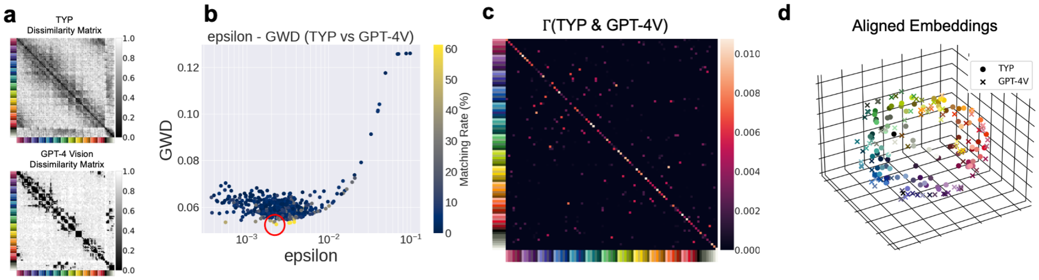

Figure S4: **Unsupervised alignment between the color similarity structure of the human color-neurotypical participants and that of GPT-4 Vision**. (a) Dissimilarity matrices of 93 colors from the human color-neurotypical participants (abbreviated by TYP) and GPT-4 Vision (abbreviated by GPT-4V). (b) The optimization results over 500 iterations with different ϵ values. GWD values of local minima represented by points are shown with respect to ϵ. Colors represent the matching rate of unsupervised alignment. (c) Optimal transportation plan Γ between the dissimilarity matrices of TYP and GPT-4 Vision. (d) Aligned embeddings of TYP and GPT-4 Vision plotted in the embedded space of TYP.

Supplementary Figure S5

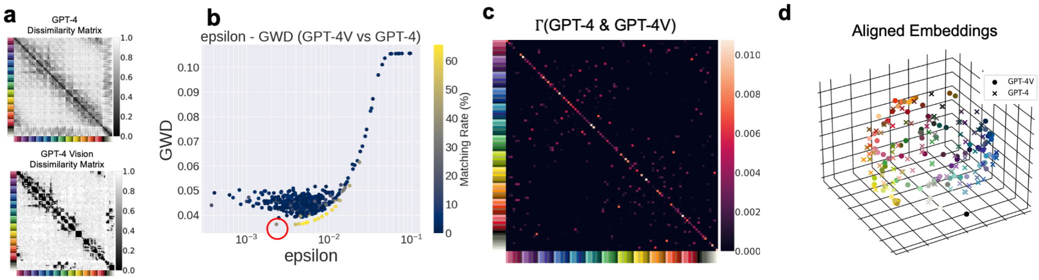

Figure S5: **Unsupervised alignment between the color similarity structure of GPT-4 and that of GPT-4 Vision**. Format is the same as Figure S4.

Supplementary Movies S1

The animations of 3D MDS embeddings for the color-neurotypical participants, the color-atypical participants, GPT-4, GPT-3.5, and the RGB and LAB color spaces viewed from different angles are available at this link. The animations of Fig. 4d (aligned embeddings of color-neurotypical participants and GPT-4), Fig. 5d (aligned embeddings of color-neurotypical participants and GPT-3.5), and Fig. 6d (aligned embeddings of color-neurotypical participants and LAB color space) are also available. Subtle structural differences between human participants, GPTs, and color space models can be visually confirmed by close inspection.

References

[1] Genji Kawakita, Ariel Zeleznikow-Johnston, Ken Takeda, Naotsugu Tsuchiya, and Masafumi Oizumi. Is my "red" your "red"?: Unsupervised alignment of qualia structures via optimal transport. January 2023.

[2] Gunnar P Epping, Elizabeth L Fisher, Ariel Zeleznikow-Johnston, Emmanuel Pothos, and Naotsugu Tsuchiya. A quantum geometric model of color similarity judgements. December 2021.

[3] Ariel Zeleznikow-Johnston, Yasunori Aizawa, Makiko Yamada, and Naotsugu Tsuchiya. Are color experiences the same across the visual field? *J. Cogn. Neurosci.*, 35(4):509–542, April 2023.

[4] J Birch. Efficiency of the ishihara test for identifying red-green colour deficiency. *Ophthalmic Physiol. Opt.*, 17(5):403–408, September 1997.

[5] Andrew Pouw, Rustum Karanjia, and Alfredo Sadun. A method for identifying color vision deficiency malingering. *Graefes Arch. Clin. Exp. Ophthalmol.*, 255(3):613–618, March 2017.

[6] Noburo Saji, Mutsumi Imai, and Michiko Asano. Acquisition of the meaning of the word orange requires understanding of the meanings of red, pink, and purple: Constructing a lexicon as a connected system. *Cogn. Sci.*, 44(1):e12813, January 2020.

[7] Raja Marjieh, Ilia Sucholutsky, Pol van Rijn, Nori Jacoby, and Thomas L Griffiths. What language reveals about perception: Distilling psychophysical knowledge from large language models. February 2023.

[8] Masaru Sasaki, Ken Takeda, Kota Abe, and Masafumi Oizumi. Toolbox for Gromov-Wasserstein optimal transport: Application to unsupervised alignment in neuroscience. September 2023.
